# Effect of perioperative intervention based on the theory of planned behavior on preventing deep vein thrombosis after laparoscopic cholecystectomy: a retrospective study

**DOI:** 10.3389/fsurg.2026.1750514

**Published:** 2026-07-07

**Authors:** Bo Wang, Xiaojun Deng, Xinguo Sun, Zhang Hu, Huiping Li, Qiong Yan

**Affiliations:** Nanhua University Affiliated Nanhua Hospital, Hengyang, Hunan, China

**Keywords:** coagulation function, laparoscopic cholecystectomy, lower extremity deep vein thrombosis, perioperative intervention, theory of planned behavior

## Abstract

**Objective:**

To assess the efficacy of a perioperative intervention based on the Theory of Planned Behavior (TPB) in preventing lower extremity deep vein thrombosis (LEDVT) and improving postoperative outcomes in patients undergoing laparoscopic cholecystectomy (LC).

**Methods:**

This retrospective study enrolled 136 gallstone patients who underwent LC at Nanhua University Affiliated Nanhua Hospital between January 2023 and January 2025. 60 patients (January 2023–December 2023) received conventional perioperative care (control group), while 76 patients (January 2024–January 2025) received TPB-based intervention (intervention group). The intervention targeted attitudes, subjective norms, and perceived behavioral control through preoperative health belief reinforcement, simulation training, intraoperative emotion regulation, and postoperative goal-setting. Outcomes included clinical recovery indicators, complication rates, coagulation function (fibrinogen, prothrombin time, activated partial thromboplastin time, D-dimer, platelet count), emotional status (Hospital Anxiety and Depression Scale), and health behaviors (Self-Rated Abilities for Health Practices).

**Results:**

Baseline characteristics were comparable between groups (all *P* > 0.05). The intervention group showed significantly shorter times to first flatus, defecation, bowel sound recovery, first ambulation, and reduced hospital stay (all *P* < 0.001). Complication rates, including LEDVT and pulmonary infection (2.63% vs. 8.33%) were lower in the intervention group (all P<0.05). The intervention group also exhibited improved coagulation profiles, lower HADS anxiety/depression scores, and higher health behavior scores (allP<0.001).

**Conclusion:**

TPB-based perioperative intervention accelerates recovery, reduces LEDVT and complications, optimizes coagulation, alleviates negative emotions, and enhances health behaviors, supporting its integration into standard perioperative care for LC patients.

## 1 Introduction

Gallbladder diseases, predominantly cholecystitis and cholelithiasis, are common digestive disorders wordwide, imposing a significant burden on healthcare systems due to their high morbidity and the substantial surgical demand they create (1). Over the past three decades, advances in minimally invasive surgical techniques have transformed the management of these conditions, with laparoscopic cholecystectomy (LC) now considered the gold standard of care. Compared to traditional open cholecystectomy, LC offers several advantages, including reduced intraoperative blood loss, minimized surgical trauma, accelerated recovery of gastrointestinal function, and shorter hospital stays, factors that have markedly improved patient outcomes and satisfaction (2). However, despite these benefits, postoperative complications remain a significant concern, with lower extremity deep vein thrombosis (LEDVT) emerging as a potentially life-threatening event (3).

LEDVT results from a pathological triad of venous stasis, vascular endothelial injury, and hypercoagulability, factors that are exacerbated during the perioperative period of LC (4). Intraoperatively, the use of carbon dioxide (CO₂) to establish pneumoperitoneum increases intra-abdominal pressure, compressing the inferior vena cava and its tributaries; this mechanical obstruction reduces venous return from the lower extremities by up to 40%, inducing significant stasis (5, 6). Concurrently, CO₂ absorption may trigger a mild systemic inflammatory response, characterized by increased levels of proinflammatory cytokines (e.g., interleukin-6 and tumor necrosis factor-α) that disrupt endothelial integrity and activate coagulation cascades (7). Postoperatively, prolonged bed rest, pain-induced immobility, and residual hypercoagulability further perpetuate these risks. If left untreated, LEDVT can progress to pulmonary embolism (PE), a complication with a mortality rate of 6%–12% in surgical patients, or chronic venous insufficiency, which impairs long-term quality of life (8).

Conventional perioperative care for LC patients typically includes general health education, vital sign monitoring, analgesic administration, and verbal encouragement for early ambulation (9). While these measures address basic postoperative needs, they often fail to target the behavioral and psychological factors influencing patient adherence to thromboprophylactic practices (10). For instance, patients may underestimate the risk of LEDVT, lack confidence in performing recommended exercises (e.g., ankle pumps), or experience anxiety that discourages mobility, factors that undermine the effectiveness of standard care (11).

The Theory of Planned Behavior (TPB), a well-established framework in health psychology, posits that an individual's behavior is primarily driven by behavioral intention, which is shaped by three core constructs: attitude (perceived benefits/harm of the behavior), subjective norm (perceived social pressure to perform the behavior), and perceived behavioral control (perceived ability to execute the behavior) (10). In the context of perioperative care, TPB offers a structured approach to modify patient behavior by reinforcing positive attitudes toward thromboprophylaxis (e.g., emphasizing the link between early ambulation and LEDVT prevention), aligning subjective norms with clinical guidelines (e.g., involving family members in supportive care), and enhancing perceived behavioral control (e.g., providing hands-on training for bed exercises) (12). Preliminary studies have shown that TPB-based interventions improve adherence to postoperative care protocols in orthopedic and colorectal surgery (13), but their efficacy in preventing LEDVT after LC remains underexplored.

This retrospective study aims to evaluate the effect of a TPB-based perioperative intervention on LEDVT prevention in patients undergoing LC. We hypothesize that this intervention will not only reduce the incidence of LEDVT but also accelerate postoperative recovery, improve psychological well-being, and enhance health-promoting behaviors compared to conventional care. By addressing the behavioral underpinnings of thromboprophylaxis, this study seeks to provide evidence for optimizing perioperative management strategies and improving the safety of LC.

## 2 Methods

### 2.1 Study participants

This retrospective study enrolled 136 patients with gallstones who underwent surgical treatment at Nanhua University Affiliated Nanhua Hospital between January 2023 and January 2025. A protocol shift occurred at the hospital on January 1, 2024: based on cumulative clinical experience, a perioperative intervention regimen grounded in the Theory of Planned Behavior (TPB) was formally implemented. Patients were stratified into two groups according to the timing of this protocol shift:

Control group: 60 patients admitted between January 2023 and December 2023, who received conventional perioperative care before the TPB-based intervention was adopted.

Intervention group: 76 patients admitted from January 2024 onwards, who received the TPB-based perioperative intervention.

Inclusion criteria were: (1) completion of hepatobiliary surgery (laparoscopic cholecystectomy); (2) stable vital signs postoperatively; (3) complete availability of clinical data (including surgical records, laboratory results, and follow-up notes). For patients with acute cholecystitis, surgery was performed within 72 h of symptom onset (emergency setting) in accordance with current clinical guidelines, while patients with chronic cholecystitis or asymptomatic cholelithiasis underwent elective laparoscopic cholecystectomy after routine preoperative preparation.

Exclusion criteria were: (1) pre-existing functional impairment of major organs (e.g., heart, lungs, liver, kidneys) confirmed by preoperative evaluations; (2) concurrent infectious diseases (e.g., pneumonia, urinary tract infection) at admission; (3) preoperative malnutrition (defined by serum albumin <30 g/L or body mass index <18.5 kg/m^2^); (4) preoperative coagulation abnormalities (e.g., prolonged prothrombin time >15 s, activated partial thromboplastin time >45 s, or thrombocytopenia <100 × 10⁹/L); (5) pre-existing cognitive impairment, psychiatric disorders (e.g., schizophrenia, major depressive disorder), or communication barriers (e.g., aphasia); (6) immediate transfer to another hospital within 24 h postoperatively (resulting in incomplete follow-up).

This study employed a single-blind design, in which the outcome assessors were unaware of the study group assignments. This study has been approved by the Ethics Committee of Nanhua University Affiliated Nanhua Hospital (No.2025-nhyy-163).

### 2.2 Clinical protocols

For emergency surgery patients with acute cholecystitis, preoperative intervention was condensed into a 2-hour intensive session delivered right after the surgical decision was made and before transfer to the operating room. In line with clinical guidelines (14), this streamlined protocol preserved the core elements of the full intervention—including health belief reinforcement, behavioral intention building, and preoperative simulation training—with hands-on practice covering positional shifts (supine to sitting, bed to chair transfer) and gentle limb exercises (ankle pumps, quadriceps contractions). The program was tailored to fit the tight timeline of emergency care. For elective surgery patients, the full preoperative intervention was implemented over at least one week. Perioperative care data were retrospectively extracted from electronic medical records and nursing charts; group-specific protocols are detailed below:

#### 2.2.1 Control group (conventional perioperative care)

Preoperative care: Patients received verbal briefings on surgical procedures, potential risks (e.g., bleeding, bile leakage), and expected postoperative recovery timelines. General psychological counseling was provided to alleviate preoperative anxiety. Routine preoperative assessments included physical examination, laboratory tests (complete blood count, liver/kidney function, coagulation profile), and imaging studies (abdominal ultrasound). Patients were instructed to fast for 12 h and abstain from fluids for 6 h preoperatively; surgical site skin preparation (shaving and disinfection) was performed.

Intraoperative care: Vital signs (heart rate, blood pressure, respiratory rate, oxygen saturation) were continuously monitored. Patients were positioned to ensure surgical access and minimize discomfort; standard intraoperative fluid resuscitation was administered without volume restrictions.

Postoperative care: Vital signs were monitored every 4 h for the first 24 h postoperatively, with close observation for signs of complications (e.g., fever, abdominal distension). Analgesic drugs (e.g., non-steroidal anti-inflammatory drugs or opioids) were administered based on numerical rating scale (NRS) pain scores (≥4 points). Patients were verbally encouraged to ambulate early to prevent thrombosis; oral intake was resumed gradually (starting with clear liquids) once nausea/vomiting had resolved. Wound care education included instructions on dressing changes and recognition of infection signs (e.g., redness, swelling, purulent drainage). Discharge education covered wound care, activity restrictions, dietary guidelines (low-fat, high-fiber), and follow-up scheduling.

#### 2.2.2 Intervention group (TPB-based perioperative intervention)

On the basis of conventional care, the TPB-based intervention was delivered in three phases (preoperative, intraoperative, postoperative) to target attitude, subjective norm, and perceived behavioral control.

Preoperative intervention: ① Health belief reinforcement: Weekly one-on-one counseling sessions were conducted until the night before surgery. Using printed handbooks and visual aids (e.g., anatomical diagrams of venous thrombosis, video clips of thrombus-induced complications), clinicians explained the pathogenesis of lower extremity deep vein thrombosis (LEDVT), its risk factors in LC patients (e.g., pneumoperitoneum-induced venous stasis), and the efficacy of preventive measures. Case studies of patients with/without LEDVT and recovery success stories were shared to enhance patients’ recognition of LEDVT prevention importance. ② Behavioral intention construction: Biweekly thematic lectures and interactive workshops were co-hosted by psychologists and hepatobiliary surgeons. Interactive Q&A sessions and role-playing exercises (e.g., simulating postoperative ambulation, practicing ankle pump exercises) were used to connect theoretical knowledge with practical skills. Patients were guided to set personalized postoperative recovery goals (e.g., “ambulate for 5 min 6 h after surgery,” “complete 20 ankle pumps per hour while in bed”) using the SMART (Specific, Measurable, Achievable, Relevant, Time-bound) framework. ③ Preoperative simulation training: Daily hands-on training was provided 1 week preoperatively, focusing on posture transition (e.g., moving from supine to sitting, transferring from bed to chair) and mild limb exercises (e.g., ankle pumps, quadriceps contractions). Simulated equipment (e.g., hospital beds, walkers) and video demonstrations were used; nurses provided real-time feedback to correct incorrect movements and ensure mastery of proper techniques.

Intraoperative intervention: ④ Emotion regulation: Before and after anesthesia induction, 15-minute emotion regulation sessions were conducted. Patients were guided through deep breathing exercises (4-second inhalation, 7-second breath-holding, 8-second exhalation) and mindfulness meditation (aided by calming music or guided audio). Intraoperative psychological support (e.g., reassuring verbal communication) was provided to maintain emotional stability.

Postoperative intervention: ⑤ Goal setting: Immediately after surgery, clinicians collaborated with patients to refine recovery goals (adjusting for intraoperative findings or postoperative pain). Daily goal progress was evaluated (e.g., whether ambulation time targets were met); goals were revised if needed (e.g., reducing ambulation duration for patients with severe pain). A paper-based tracking form was used to document daily activities (e.g., ambulation frequency, exercise compliance) and recovery milestones. ⑥ Monitoring and feedback: Patients’ activity adherence (e.g., completion of prescribed exercises) and physiological responses (e.g., lower limb circumference, skin temperature) were monitored at least twice daily. Real-time feedback was provided (e.g., “increasing ankle pump frequency to 30 times per hour may improve venous return”)； electronic tools (e.g., step counters, activity logs in hospital mobile applications) were used to record data, enabling timely adjustment of intervention intensity. ⑦ Perceived behavioral control enhancement: Health education and counseling sessions were held every 3 days until discharge. Customized materials (e.g., postoperative care booklets, exercise instruction videos) were distributed. Patients’ self-efficacy was assessed using a 5-point Likert scale (“How confident are you in performing ankle pumps independently?”), and targeted support was provided (e.g., additional one-on-one training for patients with low self-efficacy). ⑧ Social support stimulation: A WeChat-based support group was established, allowing patients and their family members to access real-time advice from clinicians and share recovery experiences. Weekly updates (e.g., educational articles on LEDVT prevention, recovery progress checklists) were sent via the group or email to maintain continuity of education and patient engagement.

### 2.3 Outcome measures

#### 2.3.1 Primary outcomes

Postoperative complications: The incidence of LEDVT, pulmonary infection, and bile leakage was documented. LEDVT was diagnosed via color Doppler ultrasound (showing non-compressible venous lumens or absent blood flow) or computed tomography venography (confirming intraluminal thrombi). Pulmonary infection was diagnosed based on clinical symptoms (fever >38.5 °C, cough with purulent sputum) and chest radiography findings (pulmonary infiltrates). Bile leakage was confirmed by abnormal bilirubin levels in drainage fluid or abdominal ultrasound showing bile collections.

Coagulation parameters: Fasting venous blood (3 mL) was collected from the upper extremity the morning before surgery and the day after surgery. After centrifugation at 1,108×g for 20 min, serum was analyzed using a CA7000 automated coagulation analyzer to measure fibrinogen (Fbg), prothrombin time (PT), activated partial thromboplastin time (aPTT), platelet count (PLT), and D-dimer.

Hemorheological parameters: Fasting venous blood was collected to measure plasma viscosity, whole blood viscosity (low shear rate: 1 s⁻^1^; high shear rate: 100 s⁻^1^), and hematocrit using a hemorheology analyzer.

#### 2.3.2 Secondary outcomes

Baseline and clinical recovery indicators: Baseline data (age, sex, disease type) were extracted from medical records. Clinical recovery indicators included surgical duration, time to first flatus, time to first defecation, time to bowel sound recovery, time to first ambulation, and total hospital stay.

Emotional status: Assessed using the Hospital Anxiety and Depression Scale (HADS) before surgery and 1 month postoperatively. The scale comprises two subscales (anxiety and depression), each with 7 items scored 0–3. Higher scores indicate more severe anxiety or depression. The scores of the two subscales range from 0 to 21, with 0 to 7 being asymptomatic, 8 to 10 being suspected of having symptoms, and 11 to 21 being definitely present.

Health behavior: Evaluated using the Chinese version of the Self-Rated Abilities for Health Practices (SRAHP) before surgery and 1 month postoperatively. The scale includes four dimensions (nutrition, psychological comfort, exercise, health responsibility), each with 7 items scored 0–4. Higher scores reflect more positive health behaviors.

### 2.4 Statistical analysis

All data were analyzed using SPSS 27.0 and R (version 4.5.0) software. Normality of continuous data was tested via the Kolmogorov–Smirnov (K-S) test. Normally distributed measurement data (expressed as mean ± standard deviation, x ± s) were compared between groups using independent samples t-tests. Categorical data (expressed as n, %) were compared using the chi-square (*χ*^2^) test. We performed sensitivity analyses to account for residual confounding factors and potential detection bias (continuous variables were categorized by median), and the results showed no confounding in the baseline data. A two-tailed *P*-value <0.05 was considered statistically significant.

## 3 Results

### 3.1 Baseline characteristics and clinical outcomes of patients in the two groups

There were no statistically significant differences in baseline characteristics between the two groups, indicating comparability (Table 1). Regardingclinical recovery indicators, the intervention group showed significantly shorter times to first flatus, first defecation, bowel sound recovery, and first ambulation, as well as a shorter overall hospital stay, compared to the control group (all *P* < 0.001). No significant difference was observed in the duration of surgery between the two groups (*P* = 0.954) (Table 2).

**Table 1:** Baseline characteristics of patients in the two groups.

Characteristic	Control group (*n* = 60)	Intervention group (*n* = 76)	t/*χ*^2^ value	*P* value
Sex, *n* (%)			0.128	0.721
Male	34 (56.67)	42 (55.26)		
Female	26 (43.33)	34 (44.74)		
Age, years (x ± s)	55.12 ± 6.34	55.78 ± 6.19	0.615	0.540
Disease type, *n* (%)			0.418	0.936
Acute cholecystitis (emergency surgery)	9 (15.00)	12 (15.79)		
Chronic cholecystitis (elective surgery)	13 (21.67)	16 (21.05)		
Cholelithiasis	28 (46.67)	35 (46.05)		
Gallbladder polyps	10 (16.66)	13 (17.11)		
Preoperative comorbidities, *n* (%)			0.527	0.913
Hypertension	15 (25.00)	20 (26.32)		
Diabetes mellitus	8 (13.33)	11 (14.47)		
Hyperlipidemia	10 (16.67)	13 (17.11)		
Preoperative Fbg, g/L (x ± s)	3.42 ± 0.51	3.45 ± 0.48	0.342	0.733
Preoperative PT, s (x ± s)	11.68 ± 0.82	11.75 ± 0.79	0.471	0.639
Preoperative D-dimer, μg/L (x ± s)	215.36 ± 42.18	218.74 ± 40.52	0.468	0.640

**Table 2:** Comparison of clinical recovery indicators between the two groups.

Clinical indicator	Control group (*n* = 60) (x ± s)	Intervention group (*n* = 76) (x ± s)	t value	*P* value
Duration of surgery, min	135.26 ± 12.84	135.68 ± 12.79	0.194	0.846
Time to first flatus, h	10.12 ± 1.05	8.15 ± 0.94	11.263	<0.001
Time to first defecation, h	11.72 ± 1.23	9.58 ± 0.98	10.847	<0.001
Time to bowel sound recovery, h	6.15 ± 0.88	4.98 ± 0.75	8.329	<0.001
Time to first ambulation, h	12.45 ± 1.29	10.18 ± 1.03	11.072	<0.001
Total hospital stay, d	10.95 ± 1.17	7.06 ± 0.72	22.345	<0.001

### 3.2 Incidence and causes of lower extremity deep vein thrombosis (LEDVT) and other postoperative complications

The total incidence of postoperative complications in the intervention group was significantly lower than that in the control group (*P* < 0.001). Specifically, the incidences of LEDVT, pulmonary infection, and bile leakage in the intervention group were 1.32%, 2.63%, and 0%, respectively, while the corresponding rates in the control group were 13.33%, 8.33%, and 3.33% (all *P* < 0.05). Among the causes of LEDVT, prolonged postoperative bed rest, hypercoagulable state, and phlebitis were the three most common in both groups; however, their proportions were notably lower in the intervention group compared with the control group (Tables 3, 4).

**Table 3:** Comparison of postoperative complication incidence between the two groups [*n* (%)].

Complication type	Control group (*n* = 60)	Intervention group (*n* = 76)	χ^2^ value	*P* value
Lower extremity deep vein thrombosis (LEDVT)	8 (13.33)	1 (1.32)	7.852	0.005
Pulmonary infection	5 (8.33)	2 (2.63)	3.947	0.047
Bile leakage	2 (3.33)	0 (0.00)	2.586	0.108
Urinary retention	4 (6.67)	1 (1.32)	4.219	0.040
Wound infection	3 (5.00)	1 (1.32)	2.931	0.087
Total complications	22 (36.67)	5 (6.58)	21.739	<0.001

**Table 4:** Causes and constituent ratios of LEDVT in the two groups [*n* (%)].

Cause of LEDVT	Control group (*n* = 8)	Intervention group (*n* = 1)	χ^2^ value	*P* value
Prolonged postoperative bed rest	4 (50.00)	0 (0.00)	4.571	0.032
Hypercoagulable state	2 (25.00)	1 (100.00)	1.800	0.179
Phlebitis	1 (12.50)	0 (0.00)	0.952	0.329
Venous wall injury	1 (12.50)	0 (0.00)	0.952	0.329
Rapid intravenous infusion	0 (0.00)	0 (0.00)	-	-
Subtotal	8 (100.00)	1 (100.00)	-	-

### 3.3 Comparison of coagulation function between the Two groups

**Figure 1:**
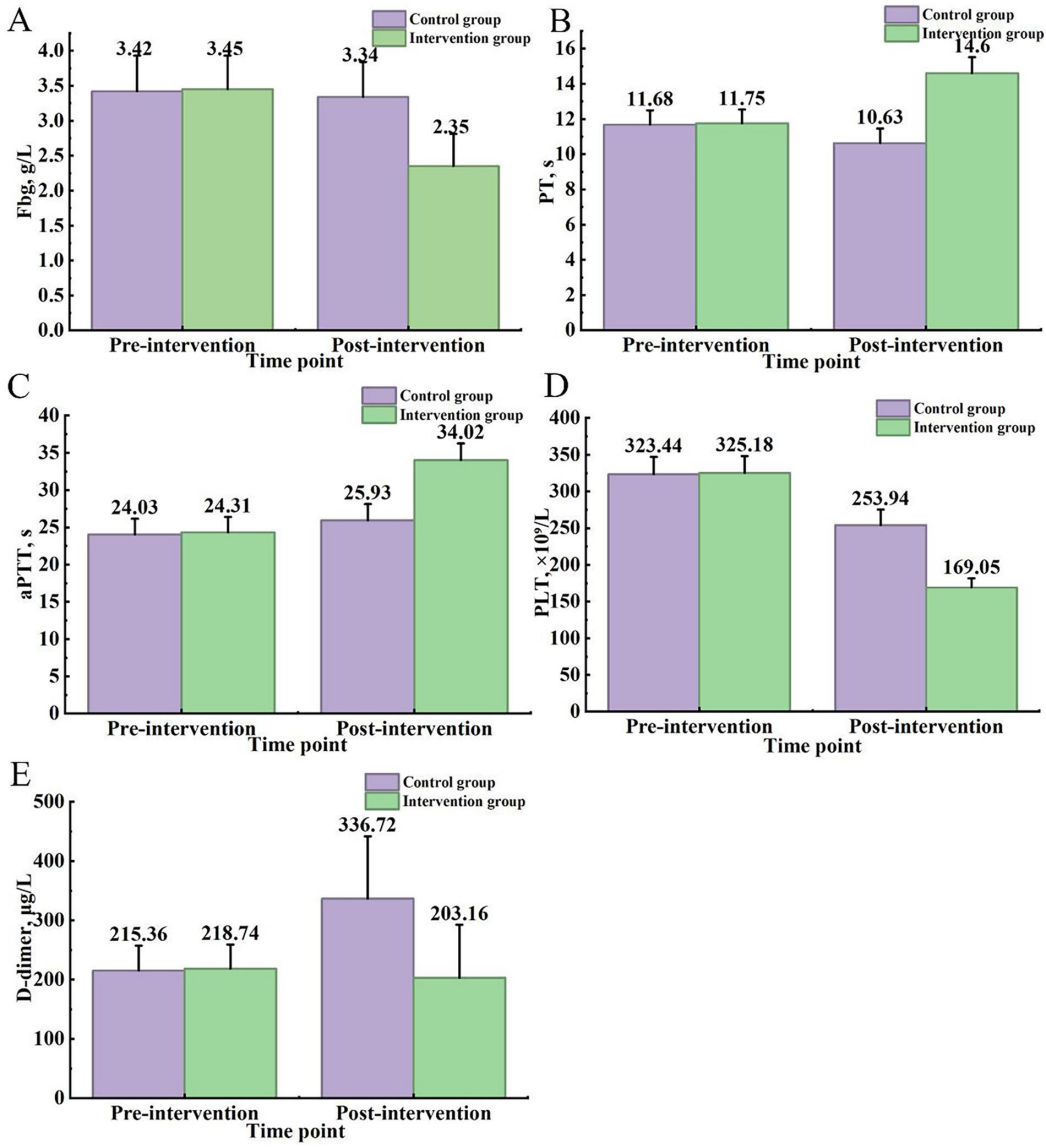
Effect of TPB-based intervention on postoperative coagulation function; panels **A–E** show the changes in various coagulation parameters pre- and post-intervention, comparing the intervention group and control group. **(A)** Fibrinogen (Fbg, g/L) levels; **(B)** prothrombin time (PT, s); **(C)** activated partial thromboplastin time (aPTT, s); **(D)** platelet count (PLT, ×10⁹/L); **(E)** D-dimer (*μ*g/L).

Before the intervention, there were no statistically significant differences in any coagulation function indicators between the two groups (all *P* > 0.05), indicating good baseline comparability. After the intervention, coagulation function improved significantly in the intervention group compared with the control group: levels of fibrinogen (Fbg), D-dimer, and platelet count (PLT) were significantly lower in the intervention group, while prothrombin time (PT) and activated partial thromboplastin time (aPTT) were significantly longer (all *P* < 0.05). In contrast, the control group only showed mild improvements in Fbg and D-dimer after intervention, with no significant changes in PT, aPTT, or PLT (Table 5; Figure 1).

**Table 5:** Comparison of coagulation function indicators between the two groups (x ± s).

Coagulation indicator	Time point	Control group (*n* = 60)	Intervention group (*n* = 76)	t value	*P* value
Fibrinogen (Fbg), g/L	Pre-intervention	3.42 ± 0.51	3.45 ± 0.48	0.342	0.733
	Post-intervention	3.34 ± 0.50	2.35 ± 0.46	11.287	<0.001
Prothrombin time (PT), s	Pre-intervention	11.68 ± 0.82	11.75 ± 0.79	0.471	0.639
	Post-intervention	10.63 ± 0.83	14.60 ± 0.91	24.153	<0.001
Activated partial thromboplastin time (aPTT), s	Pre-intervention	24.03 ± 2.11	24.31 ± 2.08	0.725	0.470
	Post-intervention	25.93 ± 2.18	34.02 ± 2.23	20.568	<0.001
Platelet count (PLT), ×10⁹/L	Pre-intervention	323.44 ± 23.56	325.18 ± 22.97	0.389	0.698
	Post-intervention	253.94 ± 21.31	169.05 ± 12.43	26.782	<0.001
D-dimer, μg/L	Pre-intervention	215.36 ± 42.18	218.74 ± 40.52	0.468	0.640
	Post-intervention	336.72 ± 105.18	203.16 ± 89.57	7.643	<0.001

### 3.4 Comparison of emotional status between the two groups

**Figure 2:**
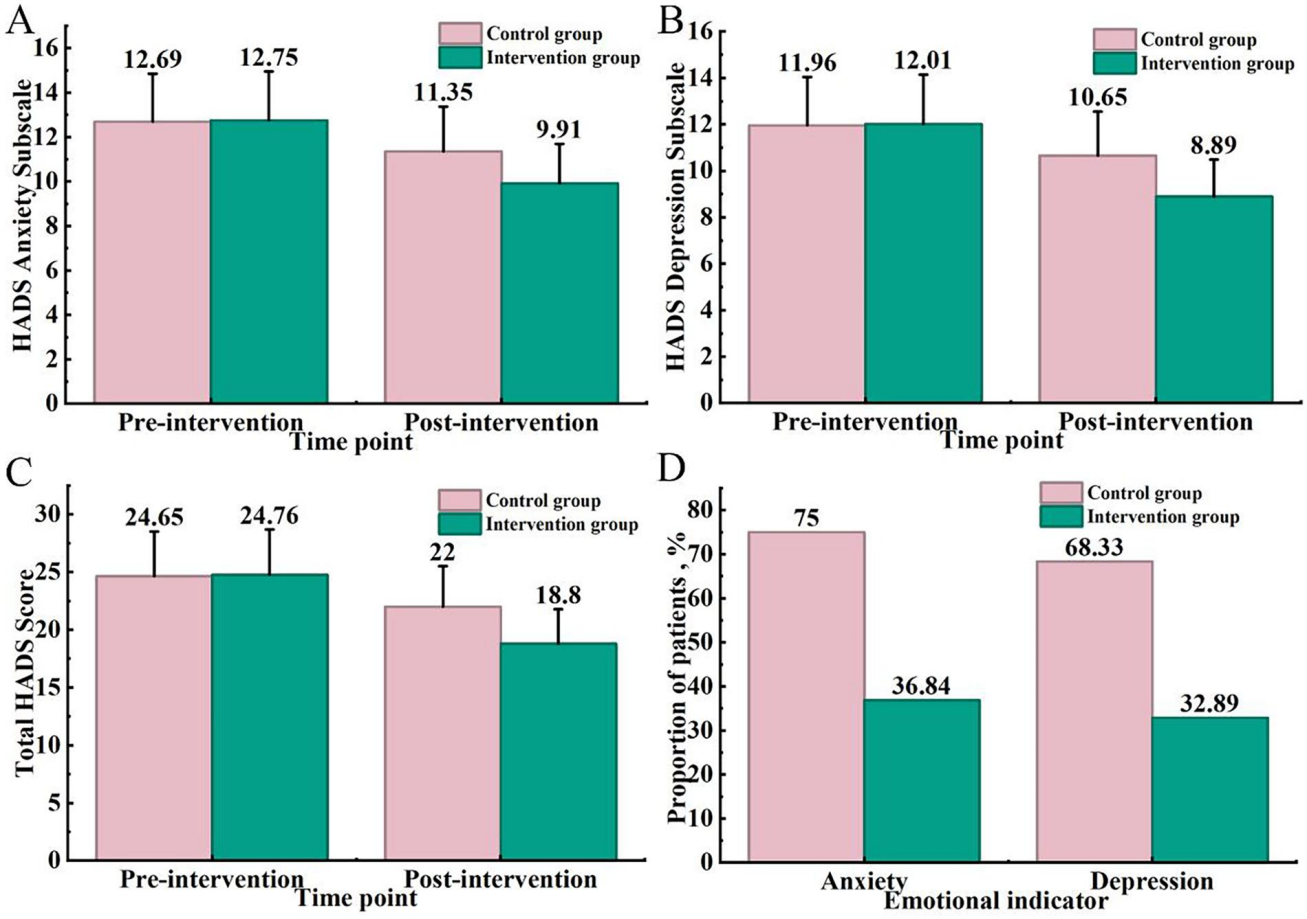
Effect of TPB-based intervention on anxiety and depression postoperatively; panels **A–D** show changes in anxiety and depression levels pre- and post-intervention, comparing the intervention group and control group. **(A)** HADS Anxiety Subscale scores; **(B)** HADS depression subscale scores; **(C)** total HADS score; **(D)** proportion of patients with anxiety and depression (percentage of patients with scores ≥8).

Before the intervention, there were no statistically significant differences in the Hospital Anxiety and Depression Scale (HADS) anxiety and depression subscale scores between the two groups (both *P* > 0.05), indicating comparable baseline emotional status. After the intervention, both groups showed reductions in HADS anxiety and depression scores, however, the decrease in the intervention group was significantly more pronounced than that in the control group. Specifically, the intervention group had significantly lower post-intervention HADS anxiety and depression scores than the control group (both *P* < 0.001). Moreover, the proportion of patients with mild-to-moderate anxiety (HADS anxiety score ≥8) or mild-to-moderate depression (HADS depression score ≥8) in the intervention group was notably lower than in the control group after the intervention (Table 6; Figure 2).

**Table 6:** Comparison of emotional status (HADS scores) between the two groups (x ± s, scores).

Emotional indicator	Time point	Control group (*n* = 60)	Intervention group (*n* = 76)	t value	*P* value
HADS Anxiety Subscale	Pre-intervention	12.69 ± 2.16	12.75 ± 2.20	0.154	0.878
	Post-intervention	11.35 ± 2.02	9.91 ± 1.78	4.218	<0.001
HADS Depression Subscale	Pre-intervention	11.96 ± 2.08	12.01 ± 2.13	0.137	0.891
	Post-intervention	10.65 ± 1.90	8.89 ± 1.59	5.532	<0.001
Total HADS Score	Pre-intervention	24.65 ± 3.87	24.76 ± 3.94	0.162	0.871
	Post-intervention	22.00 ± 3.51	18.80 ± 2.98	5.674	<0.001
Proportion of patients with anxiety (score ≥8), *n* (%)	Post-intervention	45 (75.00)	28 (36.84)	22.361	<0.001
Proportion of patients with depression (score ≥8), *n* (%)	Post-intervention	41 (68.33)	25 (32.89)	18.975	<0.001

### 3.5 Comparison of health behaviors between the two groups

**Figure 3:**
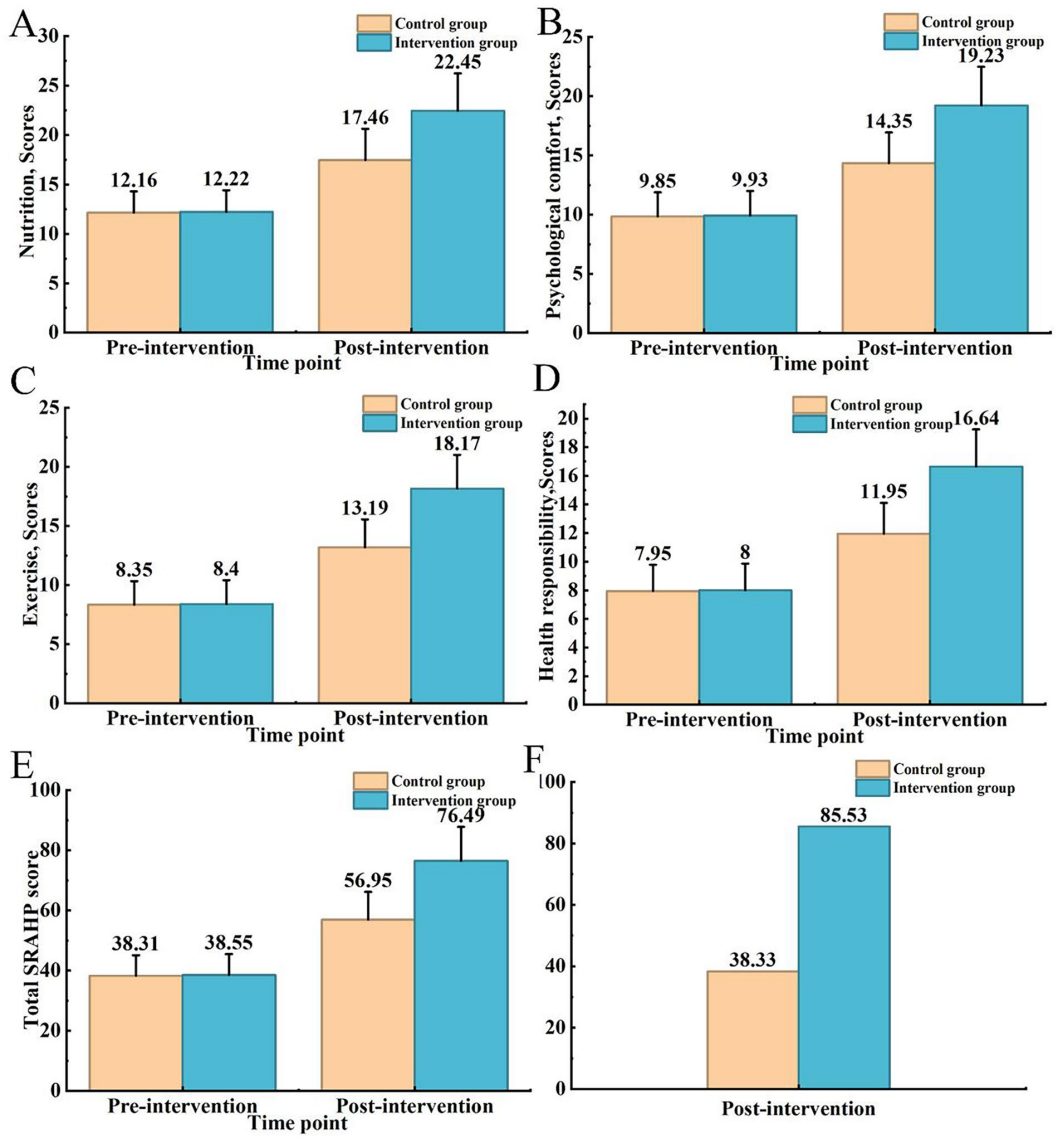
Effect of TPB-based intervention on health behaviors postoperatively; panels **A–F** show changes in health behavior scores pre- and post-intervention, comparing the intervention group and control group. **(A)** nutrition scores; **(B)** psychological comfort scores; **(C)** exercise scores; **(D)** health responsibility scores; **(E)** total self-rated abilities for health practices (SRAHP) score; **(F)** proportion of patients with high health behavior (SRAHP score ≥60).

Before the intervention, there were no statistically significant differences in the scores of all dimensions of the Chinese version of the Self-Rated Abilities for Health Practices (SRAHP) between the two groups (all *P* > 0.05), indicating consistent baseline health behavior levels. After the intervention, both groups showed increases in SRAHP scores across all dimensions, but the intervention group exhibited significantly higher scores than the control group in nutrition, psychological comfort, exercise, and health responsibility (all *P* < 0.001). Additionally, the total SRAHP score of the intervention group after the intervention was substantially higher than that of the control group, reflecting more positive overall health behaviors in the intervention group (Table 7; Figure 3).

**Table 7:** Comparison of health behaviors (SRAHP scores) between the two groups (x ± s, scores).

Health behavior dimension	Time point	Control group (*n* = 60)	Intervention group (*n* = 76)	t value	*P* value
Nutrition	Pre-intervention	12.16 ± 2.14	12.22 ± 2.20	0.157	0.875
	Post-intervention	17.46 ± 3.17	22.45 ± 3.79	8.219	<0.001
Psychological comfort	Pre-intervention	9.85 ± 2.04	9.93 ± 2.08	0.218	0.828
	Post-intervention	14.35 ± 2.59	19.23 ± 3.26	9.763	<0.001
Exercise	Pre-intervention	8.35 ± 1.99	8.40 ± 2.02	0.142	0.887
	Post-intervention	13.19 ± 2.37	18.17 ± 2.85	10.982	<0.001
Health responsibility	Pre-intervention	7.95 ± 1.83	8.00 ± 1.87	0.151	0.880
	Post-intervention	11.95 ± 2.16	16.64 ± 2.60	11.547	<0.001
Total SRAHP score	Pre-intervention	38.31 ± 6.82	38.55 ± 6.94	0.198	0.843
	Post-intervention	56.95 ± 9.24	76.49 ± 11.32	11.876	<0.001
Proportion of patients with high health behavior (total score ≥60), *n* (%)	Post-intervention	23 (38.33)	65 (85.53)	42.189	<0.001

## 4 Discussion

Laparoscopic cholecystectomy (LC) has become the treatment of choice for gallstone disease due to its minimally invasive nature; however, postoperative lower extremity deep vein thrombosis (LEDVT) remains a significant clinical concern. This retrospective study evaluated the effectiveness of a perioperative intervention based on the Theory of Planned Behavior (TPB) in 136 patients undergoing LC. The results indicated that the TPB-based intervention group demonstrated superior postoperative recovery, lower rates of LEDVT and other complications, improved coagulation function, reduced negative emotions, and better health behaviors compared to the control group, all of which support the value of integrating a behavioral psychology framework into the perioperative management of LC patients.

Compared with the control group, the intervention group showed significantly shorter times to first flatus, first bowel movement, return of bowel sounds, and first ambulation, with a 35.5% reduction in total length of hospital stay (7.06 days vs. 10.95 days) (all *P* < 0.001). These improvements may be related to the core principles of the Theory of Planned Behavior (TPB), which emphasizes attitude, subjective norm, and perceived behavioral control to promote recovery-oriented behaviors (15). Preoperative simulation training (e.g., position changes and ankle pumping exercises) provided patients with practical skills to facilitate early postoperative mobilization, while daily goal-setting and real-time feedback (e.g., tracking the frequency of getting out of bed via a pedometer) reinforced patients’ sustained engagement in recovery tasks. Unlike conventional care, where verbal encouragement often lacks specificity, TPB-based goal setting using the SMART framework (e.g., “Get out of bed and walk for 5 min 6 h post-surgery”) provides clear, achievable objectives, reduces uncertainty, and promotes active participation. Furthermore, intraoperative emotional regulation techniques (deep breathing, mindfulness meditation) may have alleviated stress-induced reduction in gastrointestinal motility, which can lead to delayed flatus passage and bowel function recovery (16). These findings are consistent with previous research indicating that behavioral interventions targeting patient agency and skill mastery can accelerate functional recovery following abdominal surgery.

The most striking finding was a 90% reduction in LEDVT incidence in the intervention group (1.32% vs. 13.33%, *P* = 0.005), accompanied by lower rates of pulmonary infection (2.63% vs. 8.33%, *P* = 0.047) and urinary retention (1.32% vs. 6.67%, *P* = 0.040), resulting in an 82.1% lower total complication rate (6.58% vs. 36.67%, *P* < 0.001). This reduction may be associated with the Thromboprophylaxis Basket (TPB)'s impact on the three pillars of thrombus formation: stasis, endothelial injury, and a hypercoagulable state (17). First, health belief reinforcement, via anatomical diagrams, complication videos, and case studies, enhanced patients’ recognition of LEDVT risk, particularly the link between prolonged bed rest and venous stasis (18). In the control group, 50% of LEDVT cases were attributed to postoperative immobility, whereas the intervention group had no LEDVT cases linked to bed rest (Table 4), reflecting improved adherence to early ambulation. Second, preoperative simulation training and postoperative monitoring of limb circumference prevented inappropriate movements that could cause venous wall injury, another key thrombotic factor (19). Third, the intervention's positive impact on coagulation function (discussed below) directly addressed hypercoagulability, a central driver of LEDVT in surgical patients (20). Notably, even the single LEDVT case in the intervention group was associated with a pre-existing hypercoagulable state (Table 4), suggesting that TPB-based care may be less effective for patients with refractory coagulation abnormalities, an observation requiring further investigation.

Postoperatively, coagulation parameters improved significantly in the intervention group compared with the control group: fibrinogen (Fbg) levels decreased by 29.6% (2.35 vs. 3.34 g/L), D-dimer levels decreased by 39.7% (203.16 vs. 336.72 μg/L), platelet count (PLT) decreased by 33.4% (169.05 vs. 253.94 × 10⁹/L), while prothrombin time (PT) and activated partial thromboplastin time (aPTT) increased by 37.4% and 31.2%, respectively (both *P* < 0.001). These changes indicate that patients transitioned from a hypercoagulable state to a more balanced state, which may be attributed to increased physical activity (21). Early ambulation and ankle pumping exercises promote venous return and reduce blood stasis. In contrast, the control group showed only slight decreases in Fbg and D-dimer levels, with no significant changes in PT, aPTT, or PLT, suggesting that verbal encouragement alone is insufficient. Since baseline coagulation parameters were comparable between the two groups (all *P* > 0.05), these differences may be attributable to the intervention. The intervention's impact on coagulation holds particular clinical significance because, in many patients undergoing laparoscopic cholecystectomy (LC), such as those with hypertension, diabetes, or advanced age, a hypercoagulable state is an unmodifiable risk factor. By addressing behavioral determinants of coagulation (such as activity levels), nursing interventions based on the Theory of Planned Behavior (TPB) complement pharmacological thromboprophylaxis (such as low-molecular-weight heparin) and mechanical measures (such as compression stockings), providing a multimodal approach to the prevention of lower extremity deep vein thrombosis (LEDVT).

This intervention may also be associated with significant psychological benefits in the intervention group: following the intervention, the Hamilton Anxiety and Depression Scale (HADS) anxiety and depression scores in the intervention group decreased by 12.7% and 16.5%, respectively (9.91 vs. 11.35; 8.89 vs. 10.65, both *P* < 0.001). The proportion of patients with mild to moderate anxiety decreased from 75.00% to 36.84% (*P* < 0.001). This effect may stem from the Theory of Planned Behavior (TPB)'s emphasis on perceived behavioral control, a concept closely linked to self-efficacy. Preoperative counseling and simulation training alleviated patients’ fears of postoperative pain or activity-related complications, while social support (such as WeChat groups with clinicians and peers) provided ongoing reassurance. In standard care, patients often experience anxiety due to uncertainty regarding recovery time or the risk of complications; TPB-based interventions address this by providing clear education [such as strategies for preventing lower extremity deep vein thrombosis (LEDVT)] and personalized goal-setting, thereby enhancing patients’ sense of control over their own recovery. Reduced anxiety and depression may also indirectly improve clinical outcomes: psychological stress triggers the release of catecholamines and cortisol, which may exacerbate a hypercoagulable state and delay the recovery of gastrointestinal function. By alleviating negative emotions, the intervention may create a “virtuous cycle” of improved mental and physical health, a hypothesis supported by the correlation between lower HADS scores in the intervention group and shorter recovery times.

The intervention group showed significant improvements across all four dimensions of the Self-Regulated Health Promotion Behavior Scale (SRAHP): nutrition (28.6% increase), psychological well-being (34.1% increase), physical activity (37.8% increase), and health responsibility (39.4% increase). Among them, 85.53% of patients achieved a “high health behavior” status (total score≥60), compared to 38.33% in the control group. This indicates that interventions based on the Theory of Planned Behavior (TPB) are associated with sustainable changes in health behaviors. For example, health belief reinforcement (such as education on low-fat, high-fiber diets) altered patients’ attitudes toward nutrition, while regular consultations (every 3 days until discharge) reinforced health responsibility by teaching patients to monitor wound healing or identify signs of infection. The social support group further reinforced subjective norms, when patients observed peers in the group adhering to healthy behaviors, they were more likely to do the same, thereby fostering a supportive community for recovery.

Studies have shown that the duration of laparoscopic cholecystectomy in Asia is significantly longer than in the Americas and Europe (22). This may be related to several clinical and institutional factors within our patient population. Compared with elective surgery, the length of hospital stay for emergency LC in cases of acute cholecystitis is significantly longer. Ahmad Zeineddin et al. (23) found that acute episodes (acute cholecystitis, common bile duct stones, or pancreatitis) are strong predictors of a complicated postoperative course (defined as a postoperative hospital stay >3 days or admission to the intensive care unit). Similarly, Kyong Joo Lee et al. (24) reported that the median length of hospital stay for patients undergoing direct cholecystectomy was 11.3 days, whereas it was 18.2 days for patients who underwent preoperative percutaneous transhepatic cholecystostomy. The prolonged hospital stay in acute cases reflects the need for preoperative stabilization, control of systemic inflammation, and a longer postoperative recovery period in the presence of gallbladder wall edema and pericholecystic inflammation (25). Furthermore, due to hospital policies, the length of hospital stay in this study includes both preoperative preparation and postoperative recovery periods. In addition, due to socioeconomic factors or patients’ own misjudgments of their condition, some patients fail to seek medical attention in a timely manner, resulting in delayed treatment. Studies have shown that delayed presentation (more than 4 days after symptom onset) independently increases the risk of postoperative complications and prolongs hospital stay (24). For elderly patients with acute suppurative cholecystitis, surgery performed more than 72 h after symptom onset is associated with prolonged postoperative hospital stay, increased blood loss, and a higher incidence of complications (26). The 2018 Tokyo Guidelines recommend that LC for acute cholecystitis should ideally be performed within 72 h of symptom onset, with a maximum window of 7–10 days (25). Consequently, these patients require a longer postoperative observation period. At the same time, preoperative comorbidities in our patients may have contributed to the prolonged hospital stay. Studies have shown that patients with higher Charlson Comorbidity Index scores have longer hospital stays following LC, particularly in emergency or urgent settings (27). This is consistent with the findings of Ahmad Zeineddin et al. (23), who reported that older age, male gender, and a higher burden of comorbidities are independent predictors of a complicated postoperative course. The average age of patients in this study (55.1–55.8 years) and the presence of multiple cardiovascular risk factors necessitated additional preoperative optimization and postoperative monitoring, thereby prolonging the total length of hospital stay.

The incidence of LEDVT was significantly lower in the intervention group; however, the incidence of LEDVT in the control group was 13.33%. This figure warrants careful interpretation, as it is significantly higher than the expected incidence reported for elective LC. We believe this high incidence may be attributable to the following factors. Our patient cohort exhibited multiple risk factors for DVT. The mean age ranged from 55.1 to 55.8 years; 36.7% of patients had acute cholecystitis, and a significant proportion had hypertension, diabetes, or hyperlipidemia, all of which are known to contribute to a hypercoagulable state (28). Emergency cholecystectomy itself is a major predictor of complications, including DVT (29). Pneumoperitoneum and patient positioning during laparotomy create a prothrombotic environment. Carbon dioxide pneumoperitoneum increases intra-abdominal pressure and reduces venous return, leading to venous stasis (30). These factors, in synergy with acute inflammation, elevate the risk of DVT to the levels observed in our control group. The control group had prolonged postoperative immobilization. Table 4 shows that 50% of LEDVT cases in the control group were directly attributable to postoperative immobilization. Prolonged bed rest, particularly venous stasis caused by acute inflammation and pneumoperitoneum, may have led to thrombus formation (31). An underlying hypercoagulable state may have been present but unrecognized. Patients in the control group had multiple DVT risk factors, which may have masked a subclinical hypercoagulable state that only manifested clinically postoperatively. Therefore, although the incidence of LEDVT in our control group was 13.33%, which is higher than the typical incidence for elective LC, this rate is reasonable given the high proportion of acute cholecystitis cases, the prothrombotic effects of pneumoperitoneum, prolonged bed rest, and multiple patient-level DVT risk factors. However, TPB-based interventions reduced this incidence by 90% to 1.32%, which is consistent with the expected DVT incidence in high-risk LC populations.

This study has several limitations. First, its retrospective, single-center design may introduce selection bias, and unmeasured factors (e.g., nurse-patient ratio differences between 2023 and 2024) cannot be fully excluded. Second, the sample size (*n* = 136) is relatively small, and the follow-up period was limited to 1 month postoperatively. At the same time, the number of DVT cases in this study was small, and longer-term data on chronic venous insufficiency or sustained health behavior changes are needed. Third, the study did not assess the relative contribution of individual TPB components (e.g., preoperative simulation vs. social support), making it difficult to identify the most impactful interventions. Future research should address these limitations by conducting multi-center, prospective randomized controlled trials with larger sample sizes and extended follow-up. Additionally, subgroup analyses (e.g., by age, comorbidity burden, or baseline coagulation status) could clarify whether TPB-based interventions are equally effective across different patient populations. Finally, integrating objective monitoring tools (e.g., wearable devices to track activity levels) and patient-reported outcome measures could also provide a more comprehensive evaluation of intervention efficacy.

In conclusion, a TPB-based perioperative intervention significantly improved postoperative recovery, reduced LEDVT and other complication, optimized coagulation function, alleviated anxiety and depression, and promoted health-enhancing behaviors in patients undergoing laparoscopic cholecystectomy. By addressing behavioral and psychological determinants of recovery, this approach complements conventional perioperative management and holds promise for improving long-term outcomes. Given its efficacy and feasibility, this intervention should be considered for integration into standard perioperative protocols for laparoscopic cholecystectomy.

## Data Availability

The original contributions presented in the study are included in the article/Supplementary Material, further inquiries can be directed to the corresponding author.
